# Development and psychometric testing of the self-regulatory questionnaire for lung cancer screening (SRQ-LCS)

**DOI:** 10.1080/08870446.2021.1879806

**Published:** 2021-02-17

**Authors:** Samantha L. Quaife, Kate E. Brain, Claire Stevens, Clara Kurtidu, Samuel M. Janes, Jo Waller

**Affiliations:** a Research Department of Behavioural Science and Health, University College London, London, UK; b Wolfson Institute of Preventive Medicine, Queen Mary University of London, London, UK; c Division of Population Medicine, Cardiff University, Cardiff, UK; d Lungs for Living Research Centre, UCL Respiratory, Division of Medicine, University College London, London, UK; e School of Cancer and Pharmaceutical Sciences, King’s College London, London, UK

**Keywords:** Lung cancer screening, screening uptake, self-regulation model, psychological, behavioural, lung cancer risk

## Abstract

**Background:**

Research implicates psychological factors in low uptake of lung cancer screening. We developed and psychometrically tested a standardised measure of these psychological determinants in preparation for a prospective, longitudinal cohort study of screening uptake.

**Methods:**

Leventhal’s Common-Sense Model of Self-Regulation of Health and Illness provided the theoretical framework to generate the initial item pool. Items were refined during expert review and cognitive interviews which tested for face validity, redundancy, acceptability and comprehensibility. An online survey piloted the refined pool with 1500 current and former (quit ≤ 15 years) smokers aged 55–80. The response distributions, internal reliability and factor structure determined the final retained constructs. Regression analyses examined these constructs’ associations with screening intention, smoking status and demographics.

**Results:**

The final measure included seven factor-derived subscales (consequences, personal control, treatment control, illness coherence, emotional representation, behavioural response and appraisal, risk perception) with Cronbach’s alphas ranging from 0.59 to 0.91 and four single-item questions (response efficacy for smoking cessation, treatment intention, perceived stigma and lung cancer survival). Most constructs were associated with smoking status and screening intention (p’s < .05).

**Conclusions:**

The Self-Regulatory Questionnaire for Lung Cancer Screening (SRQ-LCS) is an acceptable, reliable and valid measure for investigating the psychological determinants of screening uptake.

 Supplemental data for this article can be accessed here https://doi.org/10.1080/08870446.2021.1879806.

## Introduction

Lung cancer is one of the leading causes of premature mortality in the UK and is highly socially graded (Iyen-Omofoman et al., 2011). A recent analysis estimated that 47% of lung cancer deaths in England would be avoided if all groups had the same risk as those in the highest deprivation decile (Lewer et al., 2020). Effective tobacco control measures and smoking cessation support are key to reducing incidence, but equitable early diagnosis strategies are also needed to improve survival. Screening high-risk adults (aged 55–80 with a significant and recent smoking history) for lung cancer using low-dose computed tomography (LDCT) significantly reduces mortality by finding the disease at an early and treatable stage (de Koning et al., 2020; National Lung Screening Trial Research Team et al., 2011). Consequently, screening is recommended by the US Preventive Services Task Force (USPSTF) (Moyer, 2014) and is being piloted in England through NHS England’s Targeted Lung Health Check Programme. However, socioeconomic and smoking-related inequalities in participation have consistently been observed in the UK and internationally whereby lower socioeconomic position (SEP) and current smoking status predict lower uptake (Hestbech et al., 2011; McRonald et al., 2014; National Lung Screening Trial Research Team et al., 2010). Critically, this reduces the extent to which this early diagnosis strategy is reaching those at highest risk, undermining the population impact on lung cancer mortality.

The determinants of low uptake of LDCT lung screening in high-risk groups are likely to include complex structural and environmental inequalities which determine the resources, stressors, opportunities and illness experiences that ultimately shape individual differences in beliefs about health (von Wagner et al., 2011). These structural inequalities are critical to address, but research studies implicate the resulting psychological factors as potentially modifiable targets for intervention at the individual level. For example, low SEP and current smoking status have been associated with negative perceptions and beliefs about lung cancer outcomes such as fatalism and low perceived quality of life (Patel et al., 2012; Quaife et al., 2018, 2016; Smits et al., 2018), adverse emotional and social responses to perceived risk such as cancer worry, fear and perceived stigma (Ali et al., 2015; Carter-Harris et al., 2017; Quaife et al., 2016), and low perceived personal benefit of screening such as low response efficacy of early diagnosis and treatment (Patel et al., 2012; Quaife et al., 2018, 2016; Smits et al., 2018). Limitations of these studies include cross-sectional design and heterogeneity of measures used. However, together they highlight potentially important patterns of discordance between lay understanding of lung cancer and the medical model of screening; specifically, where lung cancer is perceived as a disease for which screening is ill-suited and unlikely to be effective for long-term smokers. An example is the perception that lung cancer screening is more likely to benefit those at lower risk (younger adults with a shorter smoking history) than those at higher risk (older adults with long-term smoking histories) when the opposite is true (Moyer, 2014). Similarly, in qualitative research with high-risk groups, some participants believed that treatment for lung cancer involves removing the entire affected lung (Quaife et al., 2016). However, surgery for early stage treatment is usually targeted within one lobe. Related to this, perceptions of low efficacy have been attributed to smoking cessation in older age (Quaife et al., 2016), when smoking cessation reduces risk of lung cancer mortality at all ages (Peto et al., 2000), improves lung cancer survival (Tammemagi et al., 2004), reduces risk of recurrence (Parsons et al., 2010) and lessens post-operative complications (Mason et al., 2009).

Leventhal’s et al. (2003) Common-Sense Model of Self-Regulation of Health and Illness (SRM) provides a useful framework for conceptualising these discordant perceptions and hypothesising how they might affect screening participation. The model posits that incongruence between an individual’s understanding of an illness, their emotional response and the relevant protection behaviour, undermines motivation to engage in that behaviour. This includes an individual’s cognitive representation of the illness (such as its consequences, treatment and causes), their emotional reaction, and the behaviours available to control both the illness (e.g. screening) and emotional reaction to it (e.g. avoidance). It also includes appraisal perceptions of how well (or not) these behavioural responses work in controlling the illness or emotional response. Originally developed to understand non-adherence to medication for chronic disease, the SRM has good predictive validity across diverse types of illness (Leventhal et al., 1992; Phillips et al., 2012). There is also evidence to support its application to cancer control among high-risk groups, implicating lay representations of perceived risk (Cameron, 2008; Kelly et al., 2005), controllability (Sullivan et al., 2010) and causal beliefs (Bishop et al., 2005) in adherence to cancer prevention behaviours including colorectal cancer screening, smoking abstinence and sunscreen use.

Previous studies of the psychological determinants of lung screening participation (Ali et al., 2015; Carter-Harris et al., 2017; Patel et al., 2012; Quaife et al., 2018, 2016; Smits et al., 2018) have used heterogeneous measures, making comparisons between studies difficult. Our primary aim, therefore, was to use the SRM framework to develop and validate a standardised measure of psychological determinants of screening participation by high-risk individuals, in preparation for a prospective, longitudinal cohort study of lung cancer screening uptake behaviour. Our secondary aims were to explore the demographic and smoking-related correlates of each of the measure’s constructs, and their associations with intention to be screened.

## Materials and methods

### Item generation

**Figure 1.**
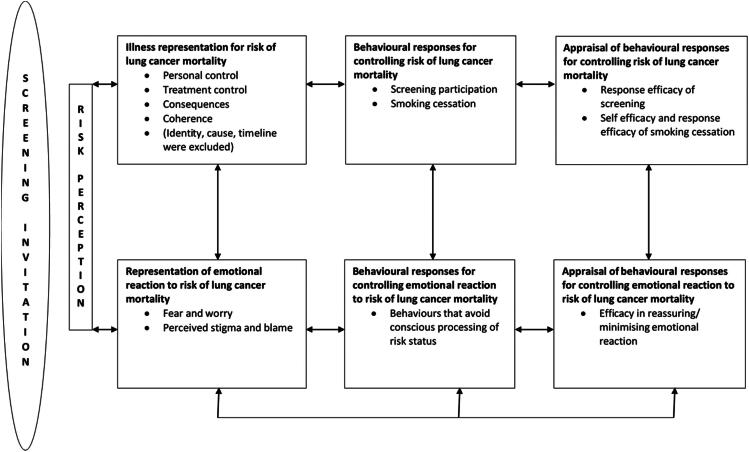
Leventhal’s et al. (2003) Common-Sense Model of Self-Regulation of Health and Illness (SRM) applied to uptake of lung cancer screening by high-risk individuals.

We used the SRM as a framework to generate the initial item pool for constructs hypothesised to affect lung cancer screening participation by high-risk asymptomatic individuals (see Figure 1). A key source of illness representation and emotional representation items was the Revised Illness Perception Questionnaire (IPQ-R (Moss-Morris et al., 2002)), which was developed to measure these constructs in the context of patients with chronic illness. This includes eight components: identity, timeline, consequences, personal control, treatment control, illness coherence, emotional representations and causes. Additional items assessing each of the SRM constructs were mostly drawn from existing electronically available research papers and surveys: risk perceptions [7 items (Ferrer et al., 2016; Kaufman et al., 2015; Lerman et al., 1993)], representation of risk of lung cancer mortality [36 items (Broadbent et al., 2006; HINTS, 2015; Moss-Morris et al., 2002; Simon et al., 2012)], behavioural responses for controlling risk of lung cancer mortality [7 items (Kotz et al., 2013)], representations of emotional reaction to risk of lung cancer mortality [15 items (Cataldo et al., 2011; Ferrer et al., 2016; Kaufman et al., 2015; Lerman et al., 1993; Marlow & Wardle, 2014; Moss-Morris et al., 2002)], behavioural responses for emotion control [16 items (Carver, 1997; HINTS, 2015; Quaife et al., 2016)], and appraisal of behavioural responses for risk and emotion [13 items, (HINTS, 2015; Jarvis et al., 2003; Joseph et al., 2018; Kaufman et al., 2015; Quaife et al., 2016; Silvestri et al., 2007)]. Minor adaptations were made to some items in order to i) ensure the disease referred to was lung cancer, ii) ensure the question was suited to both current and former smokers, or had variations to accommodate each smoking status, and iii) anchor questions to respondents’ perceptions of themselves unless the question concerned perceptions of someone with lung cancer for which items were phrased in the third person. Some original items were also created for five of the constructs (personal control, treatment control, illness coherence, emotional representations, and behavioural responses for controlling risk of lung cancer mortality). This resulted in a pool of 94 items in total.

### Item testing and refinement

A working group (n = 7) with expertise in Behavioural Science, Cancer Screening, Questionnaire Design and Tobacco Control assessed the items for face validity and potential redundancy. Cognitive interviews (n = 7) were then carried out by SLQ and CS with current and former smokers (quit ≤11 years) aged 59–71 years who had diverse educational backgrounds, including no qualifications (n = 1), CSE’s or O’levels (n = 3), further education (n = 2) and a degree (n = 1). These inclusion criteria aimed to ensure interviewees had characteristics broadly reflecting eligibility for LDCT lung cancer screening according to USPSTF criteria (Moyer, 2014), as well as varying levels of literacy. A think-aloud (Campanelli, 1997) approach was used where participants worked through each question, vocalising their responses including the reasoning behind them, any confusion or difficulties with comprehension, and any issues with acceptability. These were followed by pre-determined probes specified within a semi-structured topic guide. These probed participants about item clarity and comprehensibility, acceptability, ease of answering, suggestions for alternative item phrasing, item redundancy and preferences where there was more than one item option. Participants’ reactions to, suggestions for and perceptions of, each item were recorded informally within an unstructured matrix. Items perceived as unacceptable or unclear by at least one participant were removed or rephrased, respectively. Where there was more than one variation in phrasing for an item, the item preferred by a clear majority of participants was selected.

### Online survey

An online survey was carried out in October 2018 to pilot the refined item pool and collect data for psychometric analysis. The survey also collected information on participants’ demographic characteristics (age, gender, ethnicity, marital status) and smoking status using a question adapted from the Awareness and Beliefs about Cancer (ABC) measure (Simon et al., 2012) (‘Do you smoke cigarettes at all these days, including hand-rolled ones?). Socioeconomic position (SEP) was measured using three questions (highest level of education, housing tenure and vehicle ownership). The responses to these questions were combined to create an individual-level index ranging from zero to four; an approach used previously and shown to be associated with neighbourhood level measures of SEP and health behaviours, including cancer screening uptake (Robb et al., 2009; Wardle et al., 1999, 2004). Participants were also asked about their intentions to be screened for lung cancer (‘If you were offered a CT scan of your lungs to check for the early signs of lung cancer, would you take up the offer?’) and their willingness to have surgery in the hypothetical event that they had early stage lung cancer (‘If I had early stage lung cancer, I would want to have the recommended surgery’).

Participants were recruited by a third-party company (Research Now Survey Sampling International) from diverse panels of members of the public who had previously indicated willingness to take part in surveys. Individuals were eligible to take part if they lived in England, were aged 55–80 years and were either current or former (quit ≤15 years ago) daily tobacco smokers. A quota was applied to ensure even numbers of each smoking status were recruited. Potential participants were presented with an information screen describing the study and participants’ rights. Throughout the survey, the order of questions was rotated at random to reduce response bias due to order effects. A nominal financial incentive was provided conditional on the participant completing the survey to reduce attrition. The time taken to complete the survey was actively tracked for each participant. Data were excluded for individuals who completed the survey in an implausibly short time.

The survey was ‘soft-launched’ with a smaller sub-sample (n = 167) of participants for quality assurance purposes and to check response distributions (see Supplementary Table 1). Following further minor refinements to the wording of 14 items and the addition of 10 items which used alternative phrasing, the final survey was carried out with a larger sample (n = 1333).

**Table 1.** Demographic and smoking characteristics of the sample (n = 1333).

	All (n = 1333)	Current smokers (n = 664)	Former smokers (n = 669)
*Gender, % (n)* Female Male Prefer not to say	49.8 (664) 49.5 (660) 0.7 (9)	52.0 (345) 47.3 (314) 0.8 (5)	47.7 (319) 51.7 (346) 4 (0.6)
*Age, mean (SD)*	64.6 (6.2)	63.1 (5.8)	66.2 (6.3)
*Marital status, % (n)* Married/Civil partnership/Cohabiting Single/Divorced/Separated/Widowed	64.1 (854) 35.9 (479)	61.6 (409) 38.4 (255)	66.5 (445) 33.5 (224)
*Ethnicity, % (n)* White Minority ethnic group Prefer not to say	98.3 (1311) 1.5 (20) 0.2 (2)	97.9 (650) 2.1 (14) –	98.8 (661) 0.9 (6) 0.3 (2)
*Socioeconomic position index, % (n)* None (low SEP) One Two Three Four (high SEP) Missing	2.6 (34) 12.8 (171) 25.4 (338) 39.3 (524) 18.2 (242) 1.8 (24)	3.3 (22) 13.6 (90) 27.7 (184) 37.3 (248) 16.1 (107) 2.0 (13)	1.8 (12) 12.1 (81) 23.0 (154) 41.3 (276) 20.2 (135) 1.6 (11)
*Occupation status, % (n)* Employed (full-time/part-time/self-employed) Unemployed Home-maker Retired Not working due to long-term sickness Studying/Prefer not to say	32.3 (430) 1.8 (24) 4.9 (65) 52.9 (705) 7.9 (105) 0.3 (4)	39.5 (262) 2.0 (13) 6.2 (41) 41.7 (277) 10.4 (69) 0.3 (2)	25.1 (168) 1.6 (11) 3.6 (24) 64.0 (428) 5.4 (36) 0.3 (2)
*Smoking status, % (n)* Current smoker Former smoker	49.8 (664) 50.2 (669)	– –	– –
*Age started smoking daily, mean (SD)*	16.7 (4.2)	17.0 (4.3)	16.5 (4.1)
*Age stopped smoking, mean (SD)* ^a^	–	–	50.0 (10.4)
*Tobacco consumption (current or past), mean (SD)* Cigarettes per day Grams of tobacco per week	– –	15.7 (9.0) 53.8 (31.6)	18.1 (11.4) 75.0 (55.9)
*Time to first cigarette (tobacco dependence), % (n)*^b^ Within 5 minutes 5–30 minutes 31–60 minutes 60+ minutes	– – – –	24.5 (163) 53.3 (354) 14.0 (93) 8.1 (54)	– – – –

*Notes*. Per cent totals may not sum due to rounding.

a Former smokers only (n = 669).

b Current smokers only (n = 664).

### Psychometric analyses

Four stages of psychometric analyses were carried out to statistically test item response, validity and internal reliability. First, descriptive frequencies were calculated to examine the variation in response distributions and proportions of missing data (‘don’t know’ responses). Second, the discriminant validity of each item was tested in relation to smoking status (current vs. former), SEP, and intention to take part in LDCT lung cancer screening (intenders vs. non-intenders) using chi square analysis. While we were primarily interested in a measure that predicted screening uptake, uptake has been consistently lower among those of a current smoking status and low SEP. Therefore, we wanted to prioritise items that discriminated by these factors where possible. Principal Components Analysis (PCA) using varimax rotation was run to explore the validity of each proposed construct and to reduce the number of constructs, and as a consequence items, within the final measure. Items measuring smoking cessation, intention to be screened and intention to have treatment were excluded from the PCA. This was because they were intended as single item measures (intention items) or because they were predominantly answerable by either former or current smokers (smoking cessation items). Finally, the internal reliability of the items within each extracted PCA component was assessed using Cronbach’s alpha coefficients and item-total correlations.

### Criteria for item reduction

The results of the psychometric analyses informed the selection of items for the final measure. Specifically, the criteria were: 1) item must load (>0.4) exclusively on one of the extracted components from PCA, 2) maximum of three items per construct (to create a brief scale for planned prospective study), 3) item must discriminate by screening intention, and 4) the psychometric properties of individual items (response distribution, missing and discriminant validity) should be prioritised when their impact on internal reliability statistics for each component (difference in Cronbach’s alpha/item-total correlations) are minor.

### Analyses of final measure

T-Tests and ANOVA were used to explore differences in mean scores for each sub-scale by demographic factors and smoking status. Multivariable linear regression analyses were used to test whether each of the demographic characteristics and smoking status were independently associated with the individual sub-scales and single-item constructs. Logistic regression analyses were then carried out to explore whether these were associated with intention to be screened for lung cancer. These included both unadjusted models and models adjusted for demographic characteristics and smoking status.

### Ethics

University College London’s Research Ethics Committee granted approval for the cognitive interviews (5210/003) and survey data collection (5210/004).

## Results

### Item testing and refinement

Of the 94 items in the initial item pool, 28 were removed after review by the expert working group due to redundancy or low face validity. Minor modifications were also made to the phrasing of some items to improve clarity and face validity. This included removing items measuring the identity, timeline and cause components. Similar to Lancastle et al.’s (2011) adapted measure for ovarian cancer risk, the identity (symptom presence) and causes components were excluded so as not to cause concern among disease-free, high-risk respondents. The timeline component was excluded because unlike symptoms of chronic illness, there are no symptoms of risk which vary over time. This left 66 items for cognitive testing. Following the cognitive interviews, 13 items were removed; including two items perceived as redundant, four items perceived to have low acceptability, and seven items which did not make sense to participants in the context of lung cancer risk. These latter seven were adapted from the brief COPE measure (Carver, 1997), which were intended to measure behavioural responses for controlling one’s emotional reaction to risk of lung cancer mortality. Further minor revisions were made to the phrasing of 17 items to improve readability. Following the survey soft launch (n = 167; Supplementary Table 1), further refinements were made to the wording of 14 items and 11 alternatively phrased versions of existing items were added which aimed to improve response distribution. This resulted in a total of 64 items to be tested.

### Online survey

#### Participant characteristics

Table 1 presents the sample characteristics overall and by smoking status. Mean age of the sample was 64.6 years (range 55–80) and there was an even split by gender and smoking status. The majority of the sample reported a White ethnic background (98.3%), being married or cohabiting (64.1%), and not being in paid employment (67.8%) predominantly due to retirement (52.9%). There was good representation across the five SEP groups, with the exception of the lowest SEP group which was merged with the next lowest SEP group for further analyses. With regards to smoking history, participants reported starting smoking at age 16.7 years on average, and (among former smokers) stopping aged 50.0. Tobacco dependence was high among current smokers, with three quarters (77.8%) reporting smoking within 30 minutes of waking.

#### Psychometric analyses: response variation and discriminant validity

Supplementary Table 2 summarises the variation in response and discriminant validity for each item among the full sample (n = 1333). Twelve of the items had a skewed response distribution indicating potential ceiling effects and 18 items had a high (≥10%) ‘don’t know’ response rate. However, nearly all (60 items) discriminated by intention to be screened for lung cancer. The majority (34 items) discriminated by smoking status with the exception of the illness coherence and emotional representation sub-scales, where only the lung cancer worry and perceived stigma items discriminated. Far fewer (10 items) discriminated by socioeconomic position.

**Table 2.** Associations between demographic factors, smoking status and sub-scale scores.

	Consequences		Personal control		Treatment control		Coherence	
	Mean (SD)	**B (95% CI)** ^a^	Mean (SD)	**B (95% CI)** ^a^	Mean (SD)	**B (95% CI)** ^a^	Mean (SD)	**B (95% CI)** ^a^
***Overall***	4.57 (1.50)	–	11.10 (1.99)	–	9.85 (2.14)	–	9.08 (2.43)	–
***Gender*** Male Female	4.62 (1.52) 4.53 (1.46)	REF −0.11 (–0.27, 0.06)	11.31 (1.89)*** 10.89 (2.05)***	REF −0.40 (–0.64, −0.16)***	9.97 (2.06) 9.72 (2.21)	REF −0.15 (–0.42, 0.12)	8.98 (2.31) 9.18 (2.55)	REF 0.21 (–0.07, 0.49)
***Age***	–	0.00 (–0.01, 0.02)	–	0.01 (–0.01, 0.03)	–	0.01 (–0.01, 0.04)	–	0.02 (–0.00, 0.05)
***Socioeconomic position*** None/One (low SEP) Two Three Four (high SEP)	4.80 (1.53)*** 4.64 (1.52)*** 4.48 (1.47)*** 4.49 (1.48)***	REF −0.09 (–0.37, 0.19) −0.25 (–0.51, 0.02) −0.21 (–0.52, 0.10)	10.76 (1.90) 11.03 (2.01) 11.19 (2.04) 11.22 (1.91)	REF 0.28 (–0.15, 0.68) 0.45 (0.07, 0.83)* 0.53 (0.09, 0.98)*	9.77 (2.24) 9.85 (2.13) 9.86 (2.14) 9.82 (2.14)	REF 0.01 (–0.44, 0.46) −0.04 (–0.47, 0.38) −0.11 (–0.61, 0.39)	8.63 (2.49) 9.19 (2.33) 9.12 (2.47) 9.18 (2.37)	REF 0.59 (0.12, 1.07)* 0.58 (0.13, 1.03)* 0.78 (0.25, 1.30)**
***Marital status*** Married/cohabiting Not married/cohabiting	4.56 (1.50) 4.61 (1.48)	REF −0.02 (–0.20, 0.17)	11.11 (2.01) 11.09 (1.95)	REF 0.12 (–0.14, 0.39)	9.90 (2.15) 9.76 (2.13)	REF −0.15 (–0.45, 0.15)	8.98 (2.42) 9.25 (2.43)	REF 0.41 (0.10, 0.73)*
***Smoking status*** Former smoker Current smoker	4.42 (1.41)*** 4.73 (1.57)***	REF 0.31 (0.14, 0.48)***	11.30 (2.02)** 10.90 (1.94)**	REF −0.32 (–0.57, −0.08)*	10.04 (2.17)* 9.66 (2.10)**	REF −0.36 (–0.64, −0.08)*	9.00 (2.41) 9.15 (2.45)	REF 0.12 (–0.17, 0.41)

* p < .05.

** p < .01.

*** p < .001.

a Models adjusted for gender, age, socioeconomic position, marital status and smoking status.

Findings from Principal Components Analysis (PCA) suggested a seven-component model, which was interpreted from both the point of inflexion on a scree plot and by retaining components with eigenvalues greater than 1 (Kaiser’s criterion, 1960). Five iterations of PCA were run, beginning with 57 items and excluding 25 items found to have either low item-to-total correlations (>0.3), low communality, or to cross-load onto more than one component. In total, 32 items loaded strongly (>0.4) onto one of the seven components and these factor loadings are presented in Table 2. Of these, a further 11 items were excluded because they contributed relatively little to the reliability of their respective sub-scale when compared with other items (as indicated by Cronbach’s alpha if deleted values and item-total correlation coefficients, see Table 2). Final item selection to create seven three-item extracted components was based on the pre-specified a priori criteria (see Methods).

#### Retained items and constructs for the final measure

Supplementary Table 3 presents the items and constructs for the final measure. The constructs measured by each of the seven subscales were interpreted to be: four illness representation components (consequences, personal control, treatment control, illness coherence), one emotional representation component, one behavioural response and appraisal component (combining illness and emotion control behaviours), and one risk perception component. Scores for each sub-scale, were created by summing responses to each item (e.g. 1 = strongly disagree, 2 = disagree, 3 = neither agree nor disagree, 4 = agree, 5 = strongly agree) using reverse coding where relevant. Higher scores denoted either higher levels of the construct (higher perceived personal control, treatment control, coherence and perceived risk) or more positive perceptions (of consequences, emotional representations, behavioural control and appraisal).

**Table 2. Continued.** 

	Emotional representation		Behavioural response		Risk perception	
	Mean (SD)	**B (95% CI)** ^a^	Mean (SD)	**B (95% CI)** ^a^	Mean (SD)	**B (95% CI)** ^a^
***Overall***	7.40 (2.60)	–		–	7.52 (2.53)	–
***Gender***						
Male	7.76 (2.61)***	REF	12.58 (1.98)	REF	7.20 (2.53)***	REF
Female	7.05 (2.54)***	−0.68 (–0.98, −.40)***	12.53 (2.02)	−0.02 (–0.25, 0.21)	7.83 (2.49)***	0.47 (0.21, 0.74)***
***Age***	–	0.01 (0.01, 0.06)**	–	–0.01 (–0.03, 0.01)	–	–0.03 (–0.05, −0.00)*
*Socioeconomic position*						
None/One (low SEP)	7.44 (2.64)	REF	12.36 (2.12)	REF	8.08 (2.51)**	REF
Two	7.39 (2.64)	−0.06 (–0.55, 0.43)	12.45 (2.03)	0.05 (-0.34, 0.43)	7.64 (2.62)**	−0.31 (–0.76, 0.13)
Three	7.47 (2.64)	0.05 (–0.41, 0.52)	12.62 (1.99)	0.16 (-0.20, 0.53)	7.24 (2.52)**	−0.54 (–0.96, −0.12)*
Four (high SEP)	7.22 (2.40)	−0.10 (–0.65, 0.44)	12.79 (1.79)	0.29 (-0.14, 0.72)	7.47 (2.39)**	−0.23 (–0.72, 0.26)
***Marital status***						
Married/cohabiting	7.37 (2.59)	REF	12.63 (1.91)	REF	7.40 (2.51)**	REF
Not married/cohabiting	7.46 (2.62)	0.21 (–0.12, 0.54)	12.44 (2.15)	−0.06 (-0.32, 0.20)	7.73 (2.56)**	0.01 (–0.28, 0.30)
***Smoking status***						
*** *** Former smoker	7.54 (2.64)	REF	12.85 (1.87)***	REF	6.36 (2.53)**	REF
Current smoker	7.27 (2.56)	−0.22 (–0.52, 0.08)	12.27 (2.09)***	−0.56 (–0.80, −0.33)***	8.69 (2.18)**	2.26 (1.99, 2.53)***

* p < .05.

** p < .01.

*** p < .001.

a Models adjusted for gender, age, socioeconomic position, marital status and smoking status.

In addition, four single item questions were retained: two which had not been included in the PCA as described in the Methods (response efficacy for smoking cessation and intention to be treated for early stage lung cancer), and two which were included in the PCA but did not load strongly (<0.4) onto one of the components (perceived stigma and lung cancer survival).

#### Associations between demographic factors, smoking status and sub-scale scores

Table 2 presents the unadjusted mean scores on each factor-derived sub-scale for each demographic and smoking status group, and the mutually adjusted linear regression coefficients. Relative to men, women had statistically significantly lower perceived personal control (M:10.89 vs. 11.31; ß: –0.40, p<.001), more negative emotional representations (M:7.05 vs. 7.76; ß: –0.68, p<.001) and higher risk perceptions (M: 7.83 vs. 7.20; ß:0.47, p<.001). Older age was associated with lower perceived risk (ß: –0.03, p<.01) and more positive emotional representations (ß:0.01, p<.01). Marital status was only associated with the coherence sub-scale in adjusted analyses, with those who were not married or cohabiting reporting higher coherence than those in the married/cohabiting group (M:9.25 vs. 8.98; ß:0.41, p<.05). Higher SEP was associated with greater perceived coherence across all sub-groups (e.g. group four vs. group zero/one: M:9.18 vs. 8.63; ß:0.78, p<.01), and with increased personal control for groups three and four compared with group zero/one (M:11.22 & 11.19 vs. 10.76; ß:0.45, p<.05 & ß:0.53, p<.05). There were no consistent associations with SEP for any of the other sub-scales. Smoking status was associated with scores on five of the seven sub-scales. Compared with former smokers, current smokers perceived more positive consequences (M:4.73 vs. 4.42; ß:0.31, p<.001), lower personal control (M:10.90 vs. 11.30; ß: –0.32, p<.05), lower treatment control (M:9.66 vs. 10.04; ß: –0.36, p<.05), more negative appraisal of behavioural responses (M:12.27 vs. 12.85; ß: –0.56, p<.01), and higher risk perceptions (M:8.69 vs. 6.36; ß:2.26, p<.001).

#### Associations between subscales and lung cancer screening intention

With the exception of coherence and perceived stigma, all the constructs were statistically significantly associated with intention to be screened in analyses adjusted for demographic factors and smoking status (see Table 3). More positive perceptions of the consequences of lung cancer and the chance of survival, as well as the efficacy of early diagnosis and smoking cessation as behavioural responses, were associated with higher odds of intending to take part in screening (all p’s <.001). Similarly, higher perceived risk and perceived control (both personal and treatment) statistically significantly increased the likelihood of intending to be screened (all p’s <.001). Willingness to be treated for early stage lung cancer had the largest positive effect estimate (aOR: 10.95; 95% CI: 7.05–17.02). Conversely, more positive emotional representations of lung cancer were associated with reduced odds of holding positive screening intentions (aOR: 0.85; 95% CI: 0.79–0.91).

**Table 3.** Associations between the final measure constructs and intention to be screened.

	By intention		Unadjusted		**Adjusted** ^a^	
	Intend	Do not intend	OR (95% CI)	Significance	OR (95% CI)	Significance
**Subscales, mean (SD)**						
Consequences	4.49 (1.43)	5.34 (1.85)	0.72 (0.65, 0.80)	<.001	0.73 (0.65, 0.82)	<.001
Personal control	11.20 (1.96)	10.13 (2.05)	1.28 (1.17, 1.41)	<.001	1.26 (1.14, 1.40)	<.001
Treatment control	9.97 (2.12)	8.69 (2.02)	1.32 (1.19, 1.45)	<.001	1.31 (1.19, 1.46)	<.001
Coherence	9.10 (2.45)	8.91 (2.24)	1.03 (0.96, 1.12)	.421	1.03 (0.95, 1.12)	.418
Emotional representation	7.30 (2.57)	8.36 (2.65)	0.86 (0.81, 0.92)	<.001	0.85 (0.79, 0.91)	<.001
Behavioural response	12.82 (1.76)	9.40 (1.99)	2.60 (2.21, 3.06)	<.001	2.58 (2.18, 3.05)	<.001
Risk perception	7.55 (2.53)	7.05 (2.44)	1.09 (1.00, 1.18)	<.05	1.20 (1.09, 1.32)	<.001
**Single items, % (n)**						
Response efficacy of smoking cessation	–	–	1.39 (1.18, 1.62)	<.001	1.33 (1.12, 1.57)	<.01
Perceived stigma Disagree Agree	88.5 (432) 91.1 (693)	11.5 (56) 8.9 (68)	1.00 1.32 (0.91, 1.92)	.144	1.00 1.30 (0.88, 1.91)	.185
Treatment intention ** **Disagree Agree	69.7 (152) 96.2 (908)	30.3 (66) 36 (3.8)	1.00 10.95 (7.05, 17.02)	<.001	1.00 10.15 (6.42, 16.05)	<.001
Survival from lung cancer Fair/Poor Good	89.4 (681) 95.6 (284)	10.6 (81) 4.4 (13)	1.00 2.60 (1.42, 4.74)	<.01	1.00 2.64 (1.41, 4.95)	<.01

a Models adjusted for gender, age, socioeconomic position, marital status and smoking status.

## Discussion

This paper describes the development of the first standardised measure of the psychological determinants of lung cancer screening participation by high-risk individuals: the Self-Regulatory Questionnaire for Lung Cancer Screening (SRQ-LCS). The seven factor-derived subscales share a similar factor structure to the components of the SRM on which item generation was based, with four additional single-item constructs. All items retained for the final measure had superior psychometric properties when compared with the wider item pool with regards to their comprehensibility, response distributions, discriminant validity, internal reliability and construct validity. Nearly all the SRQ-LCS constructs were associated with intention to be screened for lung cancer, suggesting good discriminant validity for tfuture prospective studies of lung cancer screening uptake behaviour.

Most of the final constructs were associated with intention to be screened and smoking status in adjusted analyses, with comparatively fewer associations with demographic characteristics. Many of these associations were largely as expected from prior literature. For example, current smokers have previously been found to hold higher lung cancer risk perceptions when compared with former smokers, including both affective and comparative dimensions of risk perceptions (Ali et al., 2015; Quaife et al., 2018; Sach & Whynes, 2009). The finding of lower perceived personal control among current smokers and those of a lower SEP is also consistent with previous evidence that these groups more frequently report fatalistic perceptions about cancer (Miles et al., 2011; Quaife et al., 2015) and an external locus of control over their health (Poortinga et al., 2008). Women also reported lower perceived personal control as well as higher risk perceptions compared with men, which is more challenging to explain. The latter may be due to the predominantly affective items within the risk perception sub-scale (e.g. ‘*How often do you worry about lung cancer?’*), with research showing that women tend to report higher cancer worry (Vrinten et al., 2014).

There were two seemingly counterintuitive associations. First, the finding that current smokers perceived the consequences of lung cancer relatively more positively than former smokers contrasts with previous studies (Quaife et al., 2018; Smits et al., 2018). One possible explanation is that the items comprising the consequences scale in the current study concern different outcomes to that of survival which has most commonly been measured. It is possible that current smokers perceive these types of outcome (such as the impact on close others) relatively less negatively. However, the absolute difference in mean scores was small and means for both groups were within the lower (more negative) range of the scale. Alternatively, it is possible that current smokers downplay the consequences of lung cancer as a form of emotion-focussed coping. The second unexpected finding is that those with a higher (more positive) emotional representation score were less likely to intend to be screened. The Principal Components Analysis showed the risk perception, consequences and emotional representation components were distinct. Nevertheless, it is possible that participants’ perceived fear, anxiety and worry in response to thinking about lung cancer partly reflects their degree of personal concern about getting the disease and the personal consequences of the disease. Those less concerned may in turn be less likely to intend to be screened. In both instances of these incongruous findings it is important to note that a central tenet of the SRM (see Figure 1) is that the relationships between constructs are bi-directional, with interactions between them explaining lung cancer screening behaviour. The individual must perceive a certain degree of risk in order for cognitive and emotional representations to be formed, and coping behaviours, to be enacted. Coping may aim to mitigate the risk of lung cancer mortality itself or may address the emotional response. In each case, appraisal will feed back into the representations and coping mechanisms. If perceived risk is too low, these reactions and responses may not be triggered. However, if perceived risk is too high, an adverse emotional response may trigger defensive processes if illness perceptions and coping behaviours are perceived negatively, which could attenuate the self-reported risk perception so that when measured, it appears low. The planned prospective, longitudinal cohort study of lung cancer screening uptake for which this measure was developed, is designed to understand these interactions over time.

There were limitations to this study that restrict generalisation beyond the present sample. While the eligibility criteria for the online survey were based on the 2013 USPSTF screening criteria, participants are likely to be less diverse than the broader screening-eligible population by virtue of the online mode. Indeed, there were very few participants in the lowest SEP group or from ethnic minority backgrounds. This further limits generalisability, especially given that those at highest risk of lung cancer are overrepresented within lower SEP groups. More diverse samples may report different structures of beliefs as has been found previously (Jonnalagadda et al., 2012). Therefore, while the SRQ-LCS’s validity was established within the present sample, as with any PCA, further research is needed to test whether the same factor structure is confirmed in other samples. A further limitation is the hypothetical nature of the outcome variable, screening intention. There exists a widely documented ‘intention-behaviour gap’ (Sheeran, 2002) whereby intentions can be an unreliable proxy for actual behaviour. Future research should test whether the SRQ-LCS is able to discriminate screening behaviour as well as screening intentions. In addition, the SRQ-LCS is focussed on measuring individual-level cognitive and affective perceptions of risk of lung cancer mortality and screening, but there are of course likely to be wider social, environmental and structural factors determining screening behaviour. Finally, all items assessing behavioural responses for controlling one’s emotional reaction to risk of lung cancer mortality (adapted from the brief COPE measure) were removed following cognitive interviewing because they lacked face validity, particularly when intended for a population of varying risk. However, this may have excluded an important construct which deserves further examination in future studies.

The SRQ-LCS appears to provide an acceptable, reliable and valid tool to measure and understand the interplay of psychological factors instrumental in determining lung cancer screening uptake. Using standardised measures should help build and progress scientific understanding in this field and ultimately provide a rigorous evidence-base from which to isolate psychological targets for intervention.

## Supplementary Material

Supplemental Material

Supplemental Material

Supplemental Material

## Data Availability

The data that support the findings of this study are available from the corresponding author, SLQ, upon reasonable request.
