# Supplementary material for: Development and psychometric testing of the self-regulatory questionnaire for lung cancer screening (SRQ-LCS)
Source: Psychol Health. 2021 Feb 17;37(2):194–210. doi: 10.1080/08870446.2021.1879806 (PMC12097803; doi:10.1080/08870446.2021.1879806)
Supplement: Supplemental Material [file GPSH_A_1879806_SM3838.docx]

**Supplementary Table 2** Summary of psychometric properties for each item and sub-scale (n=1333)

|  | **Item response** | | **Discriminant validity** | | | **PCA** | **Internal reliability** | | **Retained?**  **(scale or single item)** |
| --- | --- | --- | --- | --- | --- | --- | --- | --- | --- |
|  | **Distribution** | **Missing** | **Smoking** | **SEP** | **Intention** | **Factor loading** | **Cronbach’s alpha if deleted** | **Item-total correlation** |  |
| ***Consequences sub-scale*** |  |  |  |  |  |  | **.645** |  |  |
| **Lung cancer is a serious condition** ^a^ | Skewed | 0.9% | YES*** | NO | YES*** | .723 | .544 | .423 | Retained - scale |
| **Lung cancer has major consequences for a person's life** ^b^ | Skewed | 1.1% | YES* | NO | YES*** | .768 | .608 | .511 | Retained - scale |
| Lung cancer does not have much effect on a person's life ^b^ | Skewed | 1.6% | YES* | NO | YES*** |  |  |  | Excluded - PCA |
| Lung cancer strongly affects the way others see a person ^b^ | Acceptable | 6.2% | NO | NO | YES* |  |  |  | Excluded - PCA |
| Lung cancer has serious financial consequences ^a^ | Skewed | 7.2% | YES* | NO | YES** | .634 | .644 | .383 | Excluded - reliability |
| **Lung cancer causes difficulties for those who are close to the person who has it** ^b^ | Skewed | 2.5% | YES* | YES* | YES*** | .622 | .572 | .468 | Retained – scale |
| A diagnosis of lung cancer is a death sentence ^c^ | Normal | 7.1% | YES*** | NO | YES*** |  |  |  | Excluded - PCA |
| These days, many people with lung cancer are able to continue with their normal activities and responsibilities ^c^ | Acceptable | 15.5% | YES* | NO | YES*** |  |  |  | Excluded – PCA |
| ***Personal control sub-scale*** |  |  |  |  |  |  | **.661** |  |  |
| There are lots of things a person can do to reduce the symptoms of lung cancer ^d^ | Acceptable | 11.7% | NO | NO | YES*** |  |  |  | Excluded - PCA |
| **What a person does can determine whether their lung cancer gets better or worse** ^b^ | Acceptable | 8.0% | YES* | NO | YES*** | .707 | .589 | .590 | Retained - scale |
| There are ways a person can slow down the development of lung cancer ^e^ | Acceptable | 14.2% | YES* | NO | YES*** | .704 | .659 | .476 | Excluded - reliability |
| **What a person with lung cancer does can affect how quickly or slowly the cancer develops** ^f^ | Acceptable | 9.3% | YES* | NO | YES*** | .763 | .640 | .510 | Retained - scale |
| What a person does can affect their chance of getting lung cancer ^f^ | Skewed | 3.3% | YES** | YES** | YES*** |  |  |  | Excluded - PCA |
| What a person does can affect whether their lung cancer is found early or late ^f^ | Skewed | 6.2% | NO | YES* | YES*** |  |  |  | Excluded - PCA |
| **Nothing a person does can make their lung cancer better or worse** ^b^ | Normal | 7.6% | YES*** | NO | YES*** | .424 | .698 | .424 | Retained - scale |
| A person can influence whether their lung cancer gets better or worse ^b^ | Acceptable | 10.1% | NO | NO | YES** |  |  |  | Excluded - PCA |
| A person's actions have no effect on whether their lung cancer gets better or worse ^b^ | Normal | 7.1% | YES** | NO | YES*** |  |  |  | Excluded - PCA |

NOTE: Retained items are denoted in bold type. ITEM SOURCE: ^a^ IPQ-R item (Moss-Morris et al., 2002); ^b^ Adapted IPQ-R item; ^c^ Adapted ABC item (Simon et al., 2012); ^d^ Original item based on IPQ-R; ^e^ Adapted item from US Health Information and National Trends Survey (HINTS, 2015); ^f^ Original item; ^g^ US survey (Silvestri et al., 2007); ^h^ Adapted from authors’ previous survey ( Quaife et al., 2016); ^i^ Adapted Cancer Stigma Scale (CASS) item; ^j^ Adapted Cataldo Lung Cancer Stigma Scale (CLCSS) item (Cataldo et al., 2011); ^k^ Smoking Toolkit Study (Fidler et al., 2011); ^l^ SCALE Special Measures (National Cancer Institute, n.d.); ^m^ Adapted item from NLST (Kaufman et al., 2015); ^n^ Authors’ previous survey (Quaife et al., 2016); ^o^ Authors’ previous survey (Quaife et al., 2018); ^p^ Adapted item from TRIRISK (Ferrer et al., 2016); *p<.05, **p<.01, ***p<.001

**Supplementary Table 2 continued** Summary of psychometric properties for each item and sub-scale

|  | **Item response** | | **Discriminant validity** | | | **PCA** | **Internal reliability** | | **Retained?**  **(scale or single item)** |
| --- | --- | --- | --- | --- | --- | --- | --- | --- | --- |
|  | **Distribution** | **Missing** | **Smoking** | **SEP** | **Intention** | **Factor loading** | **Cronbach’s alpha if deleted** | **Item-total correlation** |  |
| ***Treatment control sub-scale*** |  |  |  |  |  |  | **.764** |  |  |
| There is very little that can be done to treat lung cancer ^b^ | Normal | 7.1% | YES *** | NO | YES*** |  |  |  | Excluded - PCA |
| **Treatment is very effective in curing lung cancer** ^b^ | Normal | 12.5% | YES ** | NO | YES*** | .760 | .808 | .630 | Retained - scale |
| The negative effects of lung cancer can be prevented (avoided) by treatment ^b^ | Normal | 13.1% | NO | NO | YES*** |  |  |  | Excluded - PCA |
| The negative effects of lung cancer can be prevented (avoided) by finding it early ^b^ | Skewed | 6.2% | NO | NO | YES*** | .576 | .827 | .527 | Excluded - reliability |
| **Treatment can control lung cancer** ^b^ | Normal | 10.5% | YES ** | NO | YES*** | .726 | .809 | .622 | Retained - scale |
| Treatment can cure lung cancer ^f^ | Normal | 11.1% | YES * | NO | YES *** | .800 | .799 | .671 | Excluded - reliability |
| Treatment can reduce the symptoms of lung cancer ^f^ | Acceptable | 9.9% | YES *** | NO | YES *** |  |  |  | Excluded – PCA |
| There is no treatment that can help lung cancer ^b^ | Normal | 9.8% | YES ** | NO | YES *** |  |  |  | Excluded - PCA |
| Finding lung cancer early means that treatment is more successful ^f^ | Skewed | 6.6% | YES * | YES* | YES *** | .583 | .825 | .538 | Excluded - reliability |
| **If lung cancer is found early, what is the person’s chance of surviving?** ^g^ | Normal | 20.0% | YES * | NO | YES *** |  |  |  | Retained single item |
| **When found early, lung cancer can often be cured** ^c^ | Acceptable | 15.3% | NO | NO | YES *** | .821 | .794 | .696 | Retained - scale |
| ***Illness coherence sub-scale*** |  |  |  |  |  |  | **.784** |  |  |
| The symptoms of lung cancer are puzzling to me ^a^ | Normal | 5.9% | NO | NO | YES *** |  |  |  | Excluded - PCA |
| **I know what the symptoms of lung cancer are** ^f^ | Normal | 6.6% | NO | NO | YES ** | .807 | .744 | .587 | Retained - scale |
| Lung cancer is a mystery to me ^a^ | Normal | 2.2% | NO | NO | YES ** |  |  |  | Excluded - PCA |
| **I know very little about lung cancer** ^f^ | Normal | 1.8% | NO | NO | NO | .826 | .722 | .609 | Retained - scale |
| I understand what lung cancer is ^f^ | Acceptable | 2.6% | NO | YES* | YES ** |  |  |  | Excluded - PCA |
| Lung cancer makes sense to me ^b^ | Normal | 8.8% | NO | NO | YES ** |  |  |  | Excluded - PCA |
| **I have a clear understanding of what lung cancer is** ^b^ | Normal | 3.8% | NO | YES** | YES ** | .856 | .652 | .672 | Retained - scale |
| A person with lung cancer always has symptoms before being diagnosed ^h^ | Normal | 16.7% | NO | NO | YES * |  |  |  | Excluded - PCA |
| A person with early stage lung cancer always has symptoms ^f^ | Normal | 20.0% | NO | YES* | YES ** |  |  |  | Excluded - PCA |
| Lung cancer takes several years to develop ^f^ | Normal | 22.7% | NO | NO | YES * |  |  |  | Excluded - PCA |

NOTE: Retained items are denoted in bold type. ITEM SOURCE: ^a^ IPQ-R item (Moss-Morris et al., 2002); ^b^ Adapted IPQ-R item; ^c^ Adapted ABC item (Simon et al., 2012); ^d^ Original item based on IPQ-R; ^e^ Adapted item from US Health Information and National Trends Survey (HINTS, 2015); ^f^ Original item; ^g^ US survey (Silvestri et al., 2007); ^h^ Adapted from authors’ previous survey ( Quaife et al., 2016); ^i^ Adapted Cancer Stigma Scale (CASS) item; ^j^ Adapted Cataldo Lung Cancer Stigma Scale (CLCSS) item (Cataldo et al., 2011); ^k^ Smoking Toolkit Study (Fidler et al., 2011); ^l^ SCALE Special Measures (National Cancer Institute, n.d.); ^m^ Adapted item from NLST (Kaufman et al., 2015); ^n^ Authors’ previous survey (Quaife et al., 2016); ^o^ Authors’ previous survey (Quaife et al., 2018); ^p^ Adapted item from TRIRISK (Ferrer et al., 2016); *p<.05, **p<.01, ***p<.001

**Supplementary Table 2 continued** Summary of psychometric properties for each item and sub-scale

|  | **Item response** | | **Discriminant validity** | | | **PCA** | **Internal reliability** | | **Retained?**  **(scale or single item)** |
| --- | --- | --- | --- | --- | --- | --- | --- | --- | --- |
|  | **Distribution** | **Missing** | **Smoking** | **SEP** | **Intention** | **Factor loading** | **Cronbach’s alpha if deleted** | **Item-total correlation** |  |
| ***Emotional representations sub-scale*** |  |  |  |  |  |  | **.906** |  |  |
| The thought of lung cancer makes me feel depressed ^b^ | Normal | 2.1% | NO | NO | YES*** | .800 | .908 | .752 | Excluded - reliability |
| The thought of lung cancer makes me feel upset ^b^ | Acceptable | 2.0% | NO | NO | YES*** | .835 | .902 | .804 | Excluded - reliability |
| The thought of lung cancer makes me feel angry ^b^ | Normal | 2.6% | NO | NO | YES*** | .678 | .929 | .535 | Excluded - reliability |
| **The thought of lung cancer worries me** ^b^ | Acceptable | 1.9% | YES* | NO | YES*** | .824 | .903 | .800 | Retained - scale |
| The thought of lung cancer makes me feel uncomfortable ^f^ | Acceptable | 1.7% | NO | NO | YES*** | .792 | .910 | .733 | Excluded - reliability |
| **The thought of lung cancer makes me feel anxious** ^b^ | Acceptable | 2.3% | NO | NO | YES*** | .846 | .900 | .823 | Retained - scale |
| **The thought of lung cancer makes me feel afraid** ^b^ | Acceptable | 2.6% | NO | NO | YES*** | .827 | .910 | .822 | Retained - scale |
| A person with lung cancer is NOT to blame for their condition ^i^ | Normal | 5.1% | NO | NO | YES* |  |  |  | Excluded - PCA |
| **Some people act as though it is a person's fault that they have lung cancer** ^j^ | Acceptable | 6.3% | YES* | NO | YES** |  |  |  | Retained single item |
| ***Behavioural responses early diagnosis sub-scale*** |  |  |  |  |  |  | **.592** |  |  |
| **I would benefit from a CT lung scan, which checks for the early signs of lung cancer** ^f^ | Skewed | 5.1% | NO | NO | YES*** | .671 | .544 | .366 | Retained - scale |
| A clear CT scan would stop me worrying about lung cancer ^n^ | Acceptable | 2.8% | YES* | NO | YES*** |  |  |  | Excluded - PCA |
| **If you were offered a CT scan of your lungs to check for the early signs of lung cancer, would you take up the offer?** ^o^ | Skewed | 5.9% | NO | NO | - | N/A |  |  | Retained single item |
| **If I had early stage lung cancer, I would want to have the recommended surgery** ^g^ | Skewed | 12.8% | YES*** | NO | YES*** | N/A |  |  | Retained single item |
| **I would want to know as soon as possible if I had lung cancer** ^f^ | Skewed | 3.9% | YES*** | NO | YES*** | .748 | .352 | .527 | Retained - scale |
| **I would be reluctant to get checked for lung cancer because I worry I might have it** ^e^ | Acceptable | 2.6% | YES*** | NO | YES*** | .722 | .604 | .374 | Retained - scale |
| I avoid talking about lung cancer ^n^ | Normal | 1.7% | YES*** | NO | YES*** |  |  |  | Excluded - PCA |

NOTE: Retained items are denoted in bold type. ITEM SOURCE: ^a^ IPQ-R item (Moss-Morris et al., 2002); ^b^ Adapted IPQ-R item; ^c^ Adapted ABC item (Simon et al., 2012); ^d^ Original item based on IPQ-R; ^e^ Adapted item from US Health Information and National Trends Survey (HINTS, 2015); ^f^ Original item; ^g^ US survey (Silvestri et al., 2007); ^h^ Adapted from authors’ previous survey (Quaife et al., 2016); ^i^ Adapted Cancer Stigma Scale (CASS) item; ^j^ Adapted Cataldo Lung Cancer Stigma Scale (CLCSS) item (Cataldo et al., 2011); ^k^ Smoking Toolkit Study (Fidler et al., 2011); ^l^ SCALE Special Measures (National Cancer Institute, n.d.); ^m^ Adapted item from NLST (Kaufman et al., 2015); ^n^ Authors’ previous survey (Quaife et al., 2016); ^o^ Authors’ previous survey (Quaife et al., 2018); ^p^ Adapted item from TRIRISK (Ferrer et al., 2016); *p<.05, **p<.01, ***p<.001

**Supplementary Table 2 continued** Summary of psychometric properties for each item and sub-scale

|  | **Item response** | | **Discriminant validity** | | | **PCA** | **Internal reliability** | | **Retained/Excluded** |
| --- | --- | --- | --- | --- | --- | --- | --- | --- | --- |
|  | **Distribution** | **Missing** | **Smoking** | **SEP** | **Intention** | **Factor loading** | **Cronbach’s alpha if deleted** | **Item-total correlation** |  |
| ***Risk perceptions sub-scale*** |  |  |  |  |  |  | **.747** |  |  |
| How likely do you think it is that you will get lung cancer in your lifetime? ^m^ | Acceptable | 21.2% | YES*** | NO | YES** | .799 | .797 | .675 | Excluded - reliability |
| Compared to others your age and sex, what do you think is your chance of getting lung cancer in your lifetime? ^m^ | Acceptable | 12.5% | YES*** | NO | YES*** | .786 | .805 | .638 | Excluded - reliability |
| **Compared to SMOKERS your age and sex, what do you think is your chance of getting lung cancer in your lifetime?** ^m^ | Normal | 10.6% | YES*** | YES*** | YES*** | .783 | .812 | .624 | Retained - scale |
| **How worried are you about getting lung cancer in your lifetime?** ^p^ | Normal | 3.5% | YES*** | NO | YES*** | .667 | .803 | .646 | Retained - scale |
| **How often do you worry about lung cancer?** ^m^ | Acceptable | 1.2% | YES*** | NO | YES*** | .624 | .808 | .628 | Retained - scale |
| ***Behavioural responses smoking sub-scale*** |  |  |  |  |  |  |  |  |  |
| If you decided to give up smoking for good, how high would you rate your chances of success? ^k^ | Acceptable | 5.4% | - | YES* | NO | N/A |  |  |  |
| On a scale from 1 to 10, with 1 being the lowest and 10 being the highest, how confident are you that you could stop smoking/remain smoke free if you wanted to? ^l^ | Normal | 7.8% | - | NO | NO | N/A |  |  |  |
| **In your opinion, how much would stopping smoking reduce a person’s chances of getting lung cancer?** ^m^ | Acceptable | 5.6% | YES*** | NO | YES*** | N/A |  |  | Retained single item |
| In your opinion, how much would stopping smoking reduce YOUR chances of getting lung cancer? ^m^ | Normal | 7.2% | - | NO | YES*** | N/A |  |  |  |
| In your opinion, how much did stopping smoking reduce YOUR chances of getting lung cancer? ^m^ | Acceptable | 10.0% | - | YES*** | YES*** | N/A |  |  |  |

NOTE: Retained items are denoted in bold type. ITEM SOURCE: ^a^ IPQ-R item (Moss-Morris et al., 2002); ^b^ Adapted IPQ-R item; ^c^ Adapted ABC item (Simon et al., 2012); ^d^ Original item based on IPQ-R; ^e^ Adapted item from US Health Information and National Trends Survey (HINTS, 2015); ^f^ Original item; ^g^ US survey (Silvestri et al., 2007); ^h^ Adapted from authors’ previous survey (Quaife et al., 2016); ^i^ Adapted Cancer Stigma Scale (CASS) item; ^j^ Adapted Cataldo Lung Cancer Stigma Scale (CLCSS) item (Cataldo et al., 2011); ^k^ Smoking Toolkit Study (Fidler et al., 2011); ^l^ SCALE Special Measures (National Cancer Institute, n.d.); ^m^ Adapted item from NLST (Kaufman et al., 2015); ^n^ Authors’ previous survey (Quaife et al., 2016); ^o^ Authors’ previous survey (Quaife et al., 2018); ^p^ Adapted item from TRIRISK (Ferrer et al., 2016); *p<.05, **p<.01, ***p<.001
