# Supplementary material for: Development and psychometric testing of the self-regulatory questionnaire for lung cancer screening (SRQ-LCS)
Source: Psychol Health. 2021 Feb 17;37(2):194–210. doi: 10.1080/08870446.2021.1879806 (PMC12097803; doi:10.1080/08870446.2021.1879806)
Supplement: Supplemental Material [file GPSH_A_1879806_SM3803.docx]

| **Constructs** | **Item** |
| --- | --- |
| ***Subscales*** |  |
| Consequences | Lung cancer is a serious condition ^a^ |
|  | Lung cancer has major consequences for a person's life ^b^ |
|  | Lung cancer causes difficulties for those who are close to the person who has it ^b^ |
| Personal control | What a person does can determine whether their lung cancer gets better or worse ^b^ |
|  | What a person with lung cancer does can affect how quickly or slowly the cancer develops ^f^ |
|  | Nothing a person does can make their lung cancer better or worse ^b^ |
| Treatment control | Treatment is very effective in curing lung cancer ^b^ |
|  | Treatment can control lung cancer ^b^ |
|  | When found early, lung cancer can often be cured ^c^ |
| Illness coherence | I know what the symptoms of lung cancer are ^f^ |
|  | I know very little about lung cancer ^f^ |
|  | I have a clear understanding of what lung cancer is ^b^ |
| Emotional representation | The thought of lung cancer worries me ^b^ |
|  | The thought of lung cancer makes me feel anxious ^b^ |
|  | The thought of lung cancer makes me feel afraid ^b^ |
| Behavioural response | I would benefit from a CT lung scan, which checks for the early signs of lung cancer ^f^ |
|  | I would want to know as soon as possible if I had lung cancer ^f^ |
|  | I would be reluctant to get checked for lung cancer because I worry I might have it ^e^ |
| Risk perception | Compared to SMOKERS your age and sex, what do you think is your chance of getting lung cancer in your lifetime? ^m^ |
|  | How worried are you about getting lung cancer in your lifetime? ^p^ |
|  | How often do you worry about lung cancer? ^m^ |
| ***Single items*** |  |
| Response efficacy of smoking cessation | In your opinion, how much would stopping smoking reduce a person’s chances of getting lung cancer? ^m^ |
| Perceived stigma | Some people act as though it is a person's fault that they have lung cancer ^j^ |
| Treatment intention | If I had early stage lung cancer, I would want to have the recommended surgery ^g^ |
| Survival from lung cancer | If lung cancer is found early, what is the person’s chance of surviving? ^g^ |

**Supplementary Table 3** Final SRQ-LCS constructs and items

ITEM SOURCE: ^a^ IPQ-R item (Moss-Morris et al., 2002); ^b^ Adapted IPQ-R item; ^c^ Adapted ABC item (Simon et al., 2012); ^e^Adapted item from US Health Information and National Trends Survey (HINTS, 2015); ^f^ Original item; ^g^ US survey (Silvestri et al., 2007); ^j^ Adapted Cataldo Lung Cancer Stigma Scale (CLCSS) item (Cataldo et al., 2011); ^m^ Adapted item from NLST (Kaufman et al., 2015); ^p^ Adapted item from TRIRISK (Ferrer et al., 2016)
