# Supplementary material for: Development and psychometric testing of the self-regulatory questionnaire for lung cancer screening (SRQ-LCS)
Source: Psychol Health. 2021 Feb 17;37(2):194–210. doi: 10.1080/08870446.2021.1879806 (PMC12097803; doi:10.1080/08870446.2021.1879806)
Supplement: Supplemental Material [file GPSH_A_1879806_SM0969.docx]

**Supplementary Table 1** Response frequencies and wording refinements for items included in the soft-launched survey (n=167)

|  | **Strongly agree** | **Agree** | **Neither agree nor disagree** | **Disagree** | **Strongly disagree** | **Don’t know** |
| --- | --- | --- | --- | --- | --- | --- |
| Lung cancer is a serious condition | 70.7 (118) | 25.1 (42) | 3.0 (5) | 0.0 (0) | 1.2 (2) | 0.0 (0) |
| Lung cancer has major consequences for a person's life | 50.3 (84) | 44.9 (75) | 2.4 (4) | 0.6 (1) | 1.8 (3) | 0.0 (0) |
| Lung cancer does not have much effect on a person's life | 0.6 (1) | 1.2 (2) | 2.4 (4) | 27.5 (46) | 67.7 (113) | 0.6 (1) |
| Lung cancer strongly affects the way others see a person | 12.0 (20) | 40.1 (67) | 29.9 (50) | 11.4 (19) | 3.0 (5) | 3.6 (6) |
| Lung cancer has serious financial consequences | 19.2 (32) | 37.1 (62) | 29.9 (50) | 6.6 (11) | 1.8 (3) | 5.4 (9) |
| Lung cancer causes difficulties for those who are close to the person who has it | 32.9 (55) | 52.1 (67) | 12.6 (21) | 1.2 (2) | 0.0 (0) | 1.2 (2) |
| A diagnosis of lung cancer is a death sentence | 6.6 (11) | 22.2 (37) | 29.9 (50) | 30.5 (51) | 5.4 (9) | 5.4 (9) |
| These days, many people with lung cancer **can expect** to continue with their normal activities and responsibilities* | 2.4 (4) | 28.7 (48) | 32.3 (54) | 22.2 (37) | 3.0 (5) | 11.4 (19) |
| There are lots of things a person can do to **control** the symptoms of lung cancer* | 7.2 (12) | 35.9 (60) | 32.9 (55) | 12.0 (20) | 2.4 (4) | 9.6 (16) |
| What a person does can determine whether their lung cancer gets better or worse | 12.0 (20) | 52.1 (87) | 22.2 (37) | 5.4 (9) | 1.2 (2) | 7.2 (12) |
| There are ways a person can slow down **or disrupt** the development of lung cancer* | 9.0 (15) | 43.7 (73) | 28.7 (48) | 6.0 (10) | 1.2 (2) | 11.4 (19) |
| What a person with lung cancer does can affect how quickly or slowly the cancer develops | Added for full launch | | | | | |
| What a person does can affect their chance of getting lung cancer | Added for full launch | | | | | |
| What a person does can affect whether their lung cancer is found early or late | Added for full launch | | | | | |
| **There is** nothing a person **can do to** make their lung cancer **get** better or worse* | 0.6 (1) | 16.2 (27) | 25.1 (42) | 34.1 (57) | 13.2 (22) | 10.8 (18) |
| A person **has the power to** influence whether their lung cancer gets better or worse* | 8.4 (14) | 40.1 (67) | 29.3 (49) | 10.8 (18) | 3.6 (6) | 7.8 (13) |
| A person's actions **would** have no effect on whether their lung cancer gets better or worse* | 0.6 (1) | 12.0 (20) | 26.3 (44) | 34.1 (57) | 20.4 (34) | 6.6 (11) |
| There is very little that can be done to treat lung cancer | 4.2 (7) | 22.8 (38) | 22.2 (37) | 33.5 (56) | 10.2 (17) | 7.2 (12) |
| Treatment is **effective** in curing lung cancer* | 3.0 (5) | 22.2 (37) | 40.1 (67) | 21.6 (36) | 6.0 (0) | 7.2 (12) |
| The negative effects of lung cancer can be prevented (avoided) by treatment | 6.0 (10) | 27.5 (46) | 43.1 (72) | 12.0 (20) | 2.4 (4) | 9.0 (15) |
| The negative effects of lung cancer can be prevented (avoided) by finding it early | 19.8 (33) | 50.3 (84) | 19.2 (32) | 5.4 (9) | 1.2 (2) | 4.2 (7) |
| Treatment can control lung cancer | 5.4 (9) | 36.5 (61) | 37.6 (63) | 12.6 (21) | 0.6 (1) | 7.2 (12) |
| Treatment can cure lung cancer | Added for full launch | | | | | |
| Treatment can reduce the symptoms of lung cancer | Added for full launch | | | | | |
| There is **nothing** that can help lung cancer* | 2.4 (4) | 15.0 (25) | 21.6 (36) | 40.1 (67) | 13.2 (22) | 7.8 (13) |
| Finding lung cancer early means **that it can be cured*** | 6.0 (10) | 33.5 (56) | 35.3 (59) | 13.2 (22) | 3.0 (5) | 9.0 (15) |
| **Lung cancer** can often be cured* | 3.0 (5) | 25. (42) | 38.3 (64) | 16.2 (27) | 4.8 (8) | 12.6 (21) |
|  | **Good** | **Fair** | **Poor** |  |  | **Don’t know** |
| If lung cancer is found early, what is the person’s chance of surviving? | 16.2 (27) | 54.5 (91) | 10.8 (18) |  |  | 18.6 (31) |

NOTES: See Supplementary Tables 2 and 3 for final versions of items used in SRQ-LCS and their origins; *Bold and underlined indicates text which differs to the final version of the item used in the full launch of the survey

|  | **Strongly agree** | **Agree** | **Neither agree nor disagree** | **Disagree** | **Strongly disagree** | **Don’t know** |
| --- | --- | --- | --- | --- | --- | --- |
| The symptoms of lung cancer are puzzling to me | 1.2 (2) | 15.0 (25) | 40.1 (67) | 35.3 (59) | 6.6 (11) | 1.8 (3) |
| I know what the symptoms of lung cancer are | Added for full launch | | | | | |
| Lung cancer is a mystery to me | 0.6 (1) | 9.6 (16) | 39.5 (66) | 37.1 (62) | 12.0 (20) | 1.2 (2) |
| I know very little about lung cancer | Added for full launch | | | | | |
| I **do not** understand **lung cancer*** | 1.8 (3) | 12.0 (20) | 34.7 (58) | 41.9 (70) | 9.6 (16) | 0.0 (0) |
| Lung cancer makes sense to me | 0.6 (1) | 10.2 (17) | 31.1 (52) | 41.3 (69) | 13.2 (22) | 3.6 (6) |
| I have a clear **picture or** understanding of what lung cancer is* | 4.2 (7) | 43.1 (72) | 33.5 (56) | 16.2 (27) | 2.4 (4) | 0.6 (1) |
| A person with lung cancer always has symptoms before being diagnosed | 5.4 (9) | 28.1 (47) | 25.7 (43) | 24.6 (41) | 3.0 (5) | 13.2 (22) |
| A person with early stage lung cancer always has symptoms | Added for full launch | | | | | |
| Lung cancer **develops over a period of** several years* | 7.8 (13) | 47.9 (80) | 24.6 (41) | 7.8 (13) | 0.6 (1) | 11.4 (19) |
| The thought of lung cancer makes me feel depressed | 14.4 (24) | 37.1 (62) | 24.6 (41) | 19.2 (32) | 3.0 (5) | 1.8 (3) |
| The thought of lung cancer makes me feel upset | 14.6 (24) | 39.5 (66) | 26.3 (44) | 16.8 (28) | 1.2 (2) | 1.8 (3) |
| The thought of lung cancer makes me feel angry | 6.0 (10) | 25.7 (43) | 32.3 (54) | 26.3 (44) | 8.4 (14) | 1.2 (2) |
| The thought of lung cancer **does not worry** me* | 2.4 (4) | 12.6 (21) | 21.6 (36) | 35.3 (59) | 27.5 (46) | 0.6 (1) |
| The thought of lung cancer makes me feel uncomfortable | Added for full launch | | | | | |
| The thought of lung cancer makes me feel anxious | 14.4 (24) | 41.3 (69) | 26.9 (45) | 15.6 (26) | 1.8 (3) | 0.0 (0) |
| The thought of lung cancer makes me feel afraid | 16.2 (27) | 43.7 (73) | 25.7 (43) | 10.8 (18) | 3.6 (6) | 0.0 (0) |
| A person with lung cancer is NOT to blame for their condition | 3.0 (5) | 10.2 (17) | 51.5 (86) | 28.1 (47) | 6.0 (10) | 1.2 (2) |
| Some people act as though it is a person's fault that they have lung cancer | Added for full launch | | | | | |
| I would benefit from a CT lung scan, which checks for the early signs of lung cancer | 41.9 (70) | 40.7 (68) | 10.8 (18) | 0.0 (0) | 1.8 (3) | 4.8 (8) |
| A clear CT scan would stop me worrying about lung cancer | 19.2 (32) | 43.7 (73) | 23.4 (39) | 7.8 (13) | 2.4 (4) | 4.8 (8) |
| I would want to know as soon as possible if I had lung cancer | 46.7 (78) | 35.3 (59) | 10.8 (8) | 1.8 (3) | 0.6 (1) | 4.8 (8) |
| I would be reluctant to get checked for lung cancer because I worry I might have it | 3.6 (6) | 16.8 (28) | 15.0 (25) | 31.7 (53) | 30.5 (51) | 2.4 (4) |
| I avoid talking about lung cancer | 8.4 (14) | 22.8 (38) | 39.5 (66) | 19.2 (32) | 8.4 (14) | 1.8 (3) |
| If I had early stage lung cancer, I would want to have the recommended surgery | 35.9 (60) | 34.1 (57) | 16.8 (28) | 3.0 (5) | 1.8 (3) | 8.4 (`4) |
|  | **Yes, definitely** | **Yes, probably** | **No, probably not** | **No, definitely not** |  | **Don’t know** |
| If you were offered a CT scan of your lungs to check for the early signs of lung cancer, would you take up the offer? | 59.3 (99) | 30.5 (51) | 2.4 (4) | 1.2 (2) |  | 6.6 (11) |

**Supplementary Table 1 continued** Response frequencies and wording refinements for items included in the soft-launched survey (n=167)

NOTES: See Supplementary Tables 2 and 3 for final versions of items used in SRQ-LCS and their origins; *Bold and underlined indicates text which differs to the final version of the item used in the full launch of the survey

**Supplementary Table 1 continued** Response frequencies and wording refinements for items included in the soft-launched survey (n=167)

NOTES: See Supplementary Tables 2 and 3 for final versions of items used in SRQ-LCS and their origins; *Bold and underlined indicates text which differs to the final version of the item used in the full launch of the survey; **Current smokers only; ***Former smokers only

|  | **Very unlikely** | **Unlikely** | **Neither likely nor unlikely** | **Likely** | **Very likely** | **Don’t know** |
| --- | --- | --- | --- | --- | --- | --- |
| How likely do you think it is that you will get lung cancer in your lifetime? | 5.4 (9) | 10.8 (18) | 41.3 (69) | 19.2 (32) | 7.2 (12) | 16.2 (27) |
|  | **Much lower** | **A little lower** | **About the same** | **A little higher** | **Much higher** | **Don’t know** |
| Compared to others your age and sex, what do you think is your chance of getting lung cancer in your lifetime? | 2.4 (4) | 4.2 (7) | 35.3 (59) | 28.1 (47) | 18.6 (31) | 11.4 (19) |
| Compared to SMOKERS your age and sex, what do you think is your chance of getting lung cancer in your lifetime? | 10.8 (18) | 21.0 (35) | 40.1 (67) | 10.8 (18) | 9.6 (16) | 7.8 (13) |
|  | **Not at all** | **Slightly** | **Somewhat** | **Moderately** | **Extremely** | **Don’t know** |
| How worried are you about getting lung cancer in your lifetime? | 19.2 (32) | 32.3 (54) | 31.7 (53) | 15.6 (26) | 1.2 (2) | 0.0 (0) |
| How often do you worry about lung cancer? | 19.8 (33) | 31.1 (52) | 32.9 (55) | 10.2 (17) | 4.2 (7) | 1.8 (3) |
|  | **Extremely/ very high** | **Quite high** | **Not very high** | **Low** | **Very low** | **Don’t know** |
| If you decided to give up smoking for good, how high would you rate your chances of success?** | 5.8 (5) | 4.7 (4) | 17.4 (15) | 43.0 (37) | 8.1 (7) | 9.3 (8) |
| On a scale from 1 to 10, with 1 being the lowest and 10 being the highest, how confident are you that you could stop smoking/remain smoke free if you wanted to? | Added for full launch | | | | | |
|  | **Not at all** | **Slightly** | **Somewhat** | **Moderately** | **Extremely** | **Don’t know** |
| In your opinion, how much would stopping smoking reduce a person’s chances of getting lung cancer? | 1.8 (3) | 13.8 (23) | 18.6 (31) | 22.2 (37) | 38.3 (64) | 5.4 (9) |
| In your opinion, how much would stopping smoking reduce YOUR chances of getting lung cancer?** | 4.7 (4) | 20.9 (18) | 18.6 (16) | 24.4 (21) | 25.6 (22) | 5.8 (5) |
| In your opinion, how much did stopping smoking reduce YOUR chances of getting lung cancer?*** | 7.4 (6) | 12.3 (10) | 14.8 (12) | 27.2 (22) | 25.9 (21) | 12.3 (10) |
